# Polymer-Based Wound Dressings Loaded with Essential Oil for the Treatment of Wounds: A Review

**DOI:** 10.3390/ph17070897

**Published:** 2024-07-05

**Authors:** Bruna Michele A. de B. Buriti, Pablo Luis B. Figueiredo, Marcele Fonseca Passos, Joyce Kelly R. da Silva

**Affiliations:** 1 Instituto de Ciências Exatas e Naturais, Programa de Pós-Graduação em Química, Universidade Federal do Pará, Belém 66075-110, PA, Brazil; brunamichelebrito@gmail.com; 2 Programa de Pós-Graduação em Ciências Farmacêuticas, Universidade Federal do Pará, Belém 66079-420, PA, Brazil; pablo.figueiredo@uepa.br (P.L.B.F.); cellepassos@ufpa.br (M.F.P.); 3 Programa de Pós-Graduação em Biotecnologia, Universidade Federal do Pará, Belém 66075-110, PA, Brazil

**Keywords:** volatile compounds, biopolymers, wound dressing, wound healing, antimicrobial, anti-inflammatory

## Abstract

Wound healing can result in complex problems, and discovering an effective method to improve the healing process is essential. Polymeric biomaterials have structures similar to those identified in the extracellular matrix of the tissue to be regenerated and also avoid chronic inflammation, and immunological reactions. To obtain smart and effective dressings, bioactive agents, such as essential oils, are also used to promote a wide range of biological properties, which can accelerate the healing process. Therefore, we intend to explore advances in the potential for applying hybrid materials in wound healing. For this, fifty scientific articles dated from 2010 to 2023 were investigated using the Web of Science, Scopus, Science Direct, and PubMed databases. The principles of the healing process, use of polymers, type and properties of essential oils and processing techniques, and characteristics of dressings were identified. Thus, the plants *Syzygium romanticum* or *Eugenia caryophyllata*, *Origanum vulgare*, and *Cinnamomum zeylanicum* present prospects for application in clinical trials due to their proven effects on wound healing and reducing the incidence of inflammatory cells in the site of injury. The antimicrobial effect of essential oils is mainly due to polyphenols and terpenes such as eugenol, cinnamaldehyde, carvacrol, and thymol.

## 1. Introduction

The convergence of tissue engineering, biomaterials science, and wound healing has led to significant advances in developing novel therapeutic strategies for skin injuries [1,2]. Traditional pharmaceutical treatments are no longer viable when tissues or organs are severely diseased or lost due to trauma [3]. In such cases, the option of artificial organs (including tissues) or organ transplantation arises to reconstruct these compromised tissues or organs [4,5]. Chronic wounds and tissue defects present significant clinical challenges, requiring ongoing efforts to promote effective healing and tissue regeneration [6,7,8]. Tissue engineering aims to repair, replace, maintain, or enhance the function of a specific tissue or organ [9,10].

The process of wound healing is a fascinating and intricate mechanism encompassing four distinct phases: hemostasis, inflammation, proliferation, and remodeling [11]. Coagulation factors are activated, forming a clot of platelets to minimize blood loss at the wound site (hemostasis). This is followed by an inflammatory response, characterized by the release of proteolytic enzymes and pro-inflammatory cytokines (inflammation) [12,13]. Subsequently, angiogenesis is stimulated, leading to scar formation (proliferation). Finally, the newly formed capillaries regress, and the majority of macrophages and fibroblasts undergo apoptosis (remodeling) [14,15]. In addition to these processes, a suitable sterile covering (dressing) is also crucial, providing the characteristics of skin tissue regeneration and a natural barrier to the external environment, mimicking the epithelium [16,17]. 

Biomaterials can be used to create wound dressings. Both natural and synthetic polymers have beneficial characteristics such as adjustable biodegradation rates, mechanical properties, high porosity with varying pore sizes, and a high surface-to-volume ratio based on the synthesis technique [18,19,20]. Chitosan is a polymeric, antimicrobial, antioxidant, biocompatible, and biodegradable material with low toxicity and the ability to accelerate dermal regeneration [21,22,23] usually used in biomedical areas such as wound healing and tissue engineering.

Antibacterial properties aim to reduce inflammation caused by infections, slowing the healing process [24,25,26]. One strategy to improve biological properties is to produce smart or modern dressings interspersed with essential oils (EOs), which act as bioactive agents.

Essential oils (EOs) are volatile secondary aromatic compounds characterized by the presence of phenylpropanoids and terpenoids [27]. They have antioxidant and antibacterial effects, as well as antiviral, insecticidal, analgesic, and anti-inflammatory properties [28]. In the healing process, EOs can accelerate wound closure, improve collagen deposition, and increase fibroblast proliferation [29,30].

This review, therefore, elucidates recent advances in the potential application of hybrid materials (biomaterials/EO) in the process of healing skin wounds, based on the investigation of fifty scientific articles evaluated in the following databases: Web of Science, Scopus, Science Direct, and PubMed. This study covers the principles of the healing process, the use of natural and synthetic polymers, the type and properties of essential oils and processing techniques, and the characteristics of dressings, emphasizing the chitosan biomaterial and its properties. The aim here is to provide insights into new wound treatment and tissue regeneration approaches.

## 2. Polymeric Biomaterials

Biomaterials can be understood as material designed to assume a form that can guide any therapeutic or diagnostic procedure through interactions with living systems [31] and represent a significant fraction of the products used in healthcare [32]. Among examples are biomedical devices such as biosensors [33] and blood circulation tubes [34], implantable materials (such as sutures, plates, bone substitutes, tendons, mesh, heart valves, lenses, and teeth) [35], drug delivery vehicles [36], artificial organs (such as the heart, kidney, liver, pancreas, lungs, and skin) [37], and dressings [38], among many others.

Biomaterials are classified into three main types of materials: metals, polymers, and ceramics [39]. As scaffolds, biomaterials must allow tissue growth and maturation and support cells’ physiological activities. The particularities of each depend on the type of cell in the tissue or organ to which they will be applied [40,41,42].

### 2.1. Synthetic Polymers

Polymers are macromolecules comprising the union of small repeating units called mers. They are formed by small molecules (monomers) through chemical reactions, polyaddition, or polycondensation, for example, and can be of natural or synthetic origin [43,44,45]. In biological terms, many polymers present structures similar to the extracellular matrix of the tissue to be regenerated, avoiding chronic inflammation, immunological reactions, and toxicity [46,47]. 

Synthetic polymers can be reproduced in various forms with desirable volume and surface properties. These materials’ specific advantages include the ability to tailor their mechanical properties and degradation kinetics to suit diverse applications [48,49]. For applications such as implants or controlled drug release systems, synthetic polymers that degrade hydrolytically are preferred, as their degradation is invariant from patient to patient and the implantation site [50,51]. In opposition, biopolymers have enzymatic degradation, and this mode is investigated for tissue engineering and as a substitute for the extracellular matrix [52,53].

The most used synthetic polymers as biomaterials include poly(lactic acid) (PLA), polyvinylpyrrolidone (PVP), polyvinyl alcohol (PVA), poly(ε-caprolactone) (PCL), polyurethane (PU), polyethylene glycol (PEG), polyethylene oxide (PEO), poly(L-lactide-co-caprolactone) (PLCL), carboxymethyl cellulose (CMC), and poly(vinylidene fluoride) (PVDF), among others.

PLA is a polymer derived from renewable resources approved by the Food and Drug Administration (FDA). It has become a candidate for medical applications due to its biodegradability, biocompatibility, good mechanical properties, low cost, and potential to integrate antimicrobial agents [54,55,56,57,58]. PLA also has some limitations, such as a low degradation rate, low tenacity, low crystallization rate, low heat distortion temperature, and low reactivity between hydrophobic interactions [59,60]. 

PVP is water-soluble, biocompatible, and biodegradable. It has low toxicity, high surface activity, strong adsorption capacity, and good water vapor transmission, and is impermeable to bacteria [61,62]. It is commonly used in artificial limb manufacturing, controlled drug delivery, tissue engineering, cardiovascular devices, artificial skin, blood plasma expanders, and wound dressings [63,64,65,66]. 

PVA is a hydrophilic synthetic polymer soluble in water, whose structure is mainly composed of C-C bonds, with hydroxyl and acetate groups on the sides [67,68]. It presents properties such as soft consistency when in membrane form, inherent non-toxicity, non-carcinogenicity, biodegradability, excellent biocompatibility, high surface activity, good mechanical properties, great swelling capacity in aqueous solutions, and excellent transparency and chemical resistance [69,70,71,72], being a strong candidate for the controlled release of drugs, such as dressings, artificial organs, and contact lenses [62,73,74,75,76,77,78,79,80,81,82,83,84,85,86,87,88,89,90,91].

PCL, a polyester bioabsorbable polymer with a semi-crystalline structure, is used for various medical and pharmaceutical applications, such as delivery devices and tissue regeneration [92,93,94,95,96,97,98]. PCL has good biocompatibility, low cytotoxicity in human cells, cost benefit, and ease of manufacturing. However, it has a hydrophobic surface without functional groups, making it an ineffective cell adhesion substrate. Also, due to its low stiffness, PCL as a matrix has limited biomedical applications [99]. To overcome these limitations, the combination of bioactive materials with PCL to manufacture nanofibers has emerged as a solution, leading to the creation of new membranes with superior cell proliferation properties and wound healing [100,101,102]. The introduction of functional groups to the polymer further augments its adhesive, hydrophilic, or biocompatible properties, thereby eliciting improved cellular responses and opening up intriguing potential applications [103,104].

PU is another example of a synthetic polymer also widely used in the medical field, in the form of bioadhesive fabrics, vascular stents, artificial organs, drug administration, tissue regeneration, and dressings, linked to its excellent mechanical properties and superior hydrophobicity [105,106,107,108,109,110,111,112,113]. 

When used as a dressing material, PU presents itself as an excellent option due to the external barrier it forms against bacteria on the surface of the wound and oxygen permeability, in addition to demonstrating that it is highly compatible with living organisms, not causing rejection or irritation to the wound [114]. It also promotes a moist microenvironment that favors epithelialization. Because it is transparent, it brings greater comfort to the patient, as it is unnecessary to remove it to view the wound [115,116]. However, the hydrophobic nature of this polymer is a crucial factor in wound care, as it results in poor wound contact, leading to insufficient exudate adsorption and the inability to release embedded antibacterial agents [111].

PEG is a viscous, synthetic, water-soluble amphiphilic polymer that exhibits biological properties such as non-toxicity, biocompatibility, biodegradability, elastomeric nature, anti-fouling nature, transparency, and economy [117,118], making it suitable for various medical applications [119,120,121,122,123]. PEO is characterized by high water solubility, rapid hydration, non-toxicity, insensitivity to the pH of physiological fluids, and ease of production. It is non-linear, non-crosslinked, hydrophilic, biodegradable, biocompatible, non-toxic, and FDA-approved [124,125], thus making it suitable for biomedical applications [86,126,127,128,129,130,131,132]. PLCL is also a synthetic polymer with excellent mechanical properties, biocompatibility, and biodegradability [133]. The FDA has approved it for clinical applications. However, its poor wettability may make it slightly disadvantageous as a wound dressing material [134,135]. PVDF, together with the other polymers mentioned above, is a non-reactive thermoplastic synthetic polymer formed by the polymerization of vinylidene difluoride. Due to its excellent mechanical and biocompatibility properties, good processability, and long-term stability, PVDF can be used to encapsulate active substances in the biomedical field [136,137], as well as in electronic skins, wearable tactile sensors, microfluidic cell-based assays and self-powered cardiac devices, sensors, and tissue engineering [138,139,140,141].

### 2.2. Natural Polymers

Natural polymers or biopolymers are obtained from animals, plants, microorganisms (algae and fungi), and bacteria. Chemically, they have structures with monomers of amino acids, nucleotides, esters, or monosaccharides, which are covalently linked to form polysaccharides, peptides, polyesters, or polyphenols. Widely used natural polymers include sodium alginate, gelatin, collagen, hyaluronic acid, κ-carrageenan, cellulose, gum arabic, and chitosan [142].

Alginate is a low-cost biopolymer used in many biological applications due to its biocompatible, non-toxic, biodegradable, and gel-forming nature [71,143,144]. It is an anionic, water-soluble polysaccharide found in the cell walls of marine brown algae of the class *Phaeophyceae* [145,146]. 

Biological applications of alginate are in controlled drug release systems, extracellular matrix material for biological studies, scaffolds for wound healing, and tissue engineering [147]. For example, alginate films associated with rifampicin accelerated the healing process, achieving total wound healing in 14 days [148]. Alginate dressings are designed to maintain a moist environment in the wound bed, absorb exudate, and stop bleeding [149]. It should also control pain, reduce microbial contamination, and wound odor, and absorb proteinases [150,151].

Gelatin is also a water-soluble natural polymer extracted from animal by-products obtained by hydrolysis of collagen [152,153,154,155]. It has good biocompatibility, low antigenicity, biodegradability, and non-toxicity [156,157,158]. It has been widely used in the food and pharmaceutical industries as emulsifiers, hydrogels, microencapsulating agents for antioxidant and antibacterial essential oils, and films for wound dressings and food packaging [68,159,160,161,162,163,164].

Pure gelatin films, though brittle and easily degradable, are transformed into reliable tools when plasticizers are added. This composition increases flexibility and influences cellular activities such as proliferation, migration, and differentiation [165,166,167]. More importantly, it serves as a formulation stabilizer, ensuring the reliability of medical products, and as a plasma enhancer, further enhancing its value in the medical market [64,168].

Collagen, one of the main components of the extracellular matrix, is a diverse protein family, with each type having its unique location and function. It constitutes approximately 80% of the dry weight of human skin and is also found in connective tissues, such as bones, cartilage, and tendons. This protein is considered the most abundant in the human body [169]. Type I, for instance, is found in bone, tendons, skin, teeth, and ligaments, while type II is located in cartilage and intervertebral discs. Type III, on the other hand, is found in blood vessels, skin, and muscles, and type IV in the basement membrane and basal lamina [170,171,172].

Collagen is responsible for several essential functions, such as cell migration and differentiation, cellular behavior through the regulation of the extracellular matrix (ECM), synthesis of various proteins, shear resistance to absorb energy, and ductility [169,173,174]. It can also be successfully used for tissue regeneration engineering in vitro and in vivo [175,176,177,178,179].

Collagen dressings belong to bioactive dressings that are composed of collagen obtained from bovine, porcine, marine, and avian sources, among others, and are available in the form of particles, gels, pads, ropes, sheets, or solutions [180,181]. It is a dressing impermeable to bacteria. It maintains the healing process by maintaining a moist environment around the wound, absorbs exudate, and promotes the derivation of numerous cells, such as keratinocytes and fibroblasts [182].

Hyaluronic acid is a polymer present in the extracellular matrix, with viscoelastic and hygroscopic properties, and controls cell proliferation and migration [183,184,185]. Hyaluronic acid possesses biocompatibility, biodegradability, non-immunogenicity, and wound healing properties [186,187]. Hyaluronic acid’s sizable supramolecular structures can trap large amounts of water and ions to provide hydration and turgidity to tissues, thus being considered an attractive anti-fouling material [188,189]. 

Carrageenin, a linear polysaccharide derived from the sea, is highly adaptable in the field of wound healing. Its ability to mimic the microarchitecture of the extracellular matrix of living tissues, coupled with its high water absorption capacity, hemostatic nature, good biocompatibility, and hydrogel-forming ability, makes it a promising candidate [190,191]. However, the challenge of controlling its gelling properties, mechanical stability, and high degradation rate in the biological environment has limited its application as a wound dressing [192,193]. To overcome these limitations and further enhance its properties, carrageenin can be combined with other polymers or pharmacologically active agents that can stimulate wound healing at each stage [123,194,195,196,197,198].

Chitosan and gum arabic, as biopolymers, are chosen as encapsulating materials for their biocompatibility and low toxicity [80,199]. Gum arabic, a negatively charged polysaccharide–protein complex with excellent emulsifying properties, is obtained from the stems and branches of acacia trees. The hydrophilic parts of these polymers play a crucial role in stabilizing the emulsion against droplet aggregation, while the hydrophobic branched proteins are rapidly adsorbed on the emulsion droplet surface [200,201]. In addition to emulsifying properties, gum arabic’s high solubility, lower viscosity, good film formation, and non-toxicity further reinforce its biocompatibility [202,203]. 

Chitosan is a natural, semi-crystalline cationic polymer derived from chitin in fungi’s cell walls or the exoskeleton of arthropods [204]. Chitosan-based films, with their moderate oxygen barrier and good carbon dioxide barrier properties [205,206], are instrumental in maintaining a moist environment around the lesion, a critical factor in wound healing [207,208].

#### Chitosan

**Figure 1:**
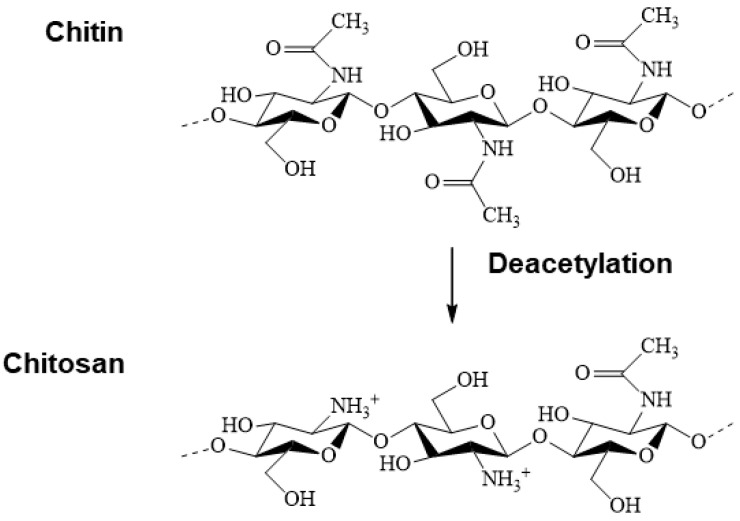
Chemical structure of chitin and chitosan.

Chitosan, discovered and named in 1859 by Roget [209], is obtained by the partial deacetylation of chitin poly(β-(1→4)-N-acetyl-D-glucosamine), under alkaline conditions, being the most crucial derivative in terms of applications [210,211,212]. Chemically, it is a copolymer of various proportions of N-acetyl-d-glucosamine and d-glucosamine (Figure 1). Its properties are highly dependent on its degree of deacetylation, average molecular weight, polydispersity, and structure [213,214].

Changes in charge density affect the dissolution and binding properties of chitosan [215,216,217]. The variation in charge density gives rise to a considerable change in the swelling index, pore size, and permeability of the chitosan drug membrane [218]. The molecular weight alters the content of N-acetylglucosamine units in chitosan, which will have both an intramolecular and intermolecular influence, resulting in different conformations [216].

Intrinsic properties of chitosan such as biocompatibility, biodegradability, non-toxicity, biological adhesiveness, antimicrobial activity, and hemostatic effect [199,216,219,220,221,222,223,224] make it a versatile material. With its reactive amino groups, chitosan is the only natural cationic polymer with numerous commercial applications: it accelerates wound healing and is an anticoagulant, antifungal, and antitumor [216,220,221,222,225]. The cationic nature of chitosan is the key to most of its biological properties, with the degree of deacetylation (DG) being the parameter with the most significant impact [226]. 

The effect of the constant molecular weight (around 810 kDa) and variable degree of deacetylation (75%, 87%, and 96%) of chitosan membranes influences tensile strength and results in more excellent elongation at break [227]. The degree of deacetylation can also affect the antimicrobial activity of chitosan [228,229], where the high DG increases the electrostatic binding to the cell membrane and the permeabilizing effect. On the other hand, the high molecular weight generates high permeation in the cell nucleus [230,231,232,233]. 

The various studies on the toxicity of chitosan [82,234,235,236,237,238,239] have reported its potential use as a biocompatible biomaterial. Chitosan/gelatin scaffolds loaded with an ethanolic extract of *Jatropha mollissima* (EEJM) and chitosan/bioglass composites tested with mouse fibroblasts and L929 fibroblasts by MTT, respectively, were shown to be non-cytotoxic. Although not present in mammals, chitosan is subject to in vivo degradation by many enzymes such as pepsin, lysozyme, and papain [240,241].

The biodegradation kinetics of chitosan is closely linked to the degree of crystallinity, primarily influenced by the degree of deacetylation [224,232]. As the degree of deacetylation decreases, biodegradation increases [232,242,243,244]. The impact of chain length (molecular weight) on the biodegradation rate has also been well documented [245,246]. Controlling the degradation rate of chitin- and chitosan-based devices is highly desirable since biodegradation is crucial for small and large molecule release applications and regeneration functional tissue applications [232].

The ease of modifying chitosan with other bioactive molecules can provide additional properties for tissue construction [247]. Chitosan membranes/films, hydrogels, sponges, scaffolds, and fibers have been investigated for wound healing or tissue engineering applications [247,248,249], as excipients for drug administration [250], and in gene delivery [251,252].

In tissue engineering, chitosan plays a pivotal role in positively influencing the different phases of the wound healing process [209,253,254]. Chitosan’s ability to modulate the activation of platelets, promote blood clotting in vivo, and regulate the activity of inflammatory cells creates a promising microenvironment for healing. It also provides non-protein matrix support for tissue growth [255]. Furthermore, chitosan’s gradual depolymerization to release N-acetyl-β-D-glucosamine stimulates fibroblast proliferation, angiogenesis, and orderly collagen deposition at the wound site [209,253,256]. On top of these benefits, chitosan can also prevent skin infections, a significant complication associated with wound healing [26].

Despite its numerous favorable properties, chitosan, on the other hand, has poor mechanical properties. To this end, to improve these properties, blends and mixtures of chitosan with other synthetic or natural polymers have been used [78,79,83,130,257]. Furthermore, studies have demonstrated the bacteriostatic and bactericidal effects of chitosan, its possible mechanisms of action against pathogenic microorganisms [258], and the influence of molecular mass, source of origin of this biopolymer, degree of deacetylation, type of microorganism, and cultivation, in biological properties. During the wound healing process, chitosan and its derivatives have also acted efficiently in the hemostasis, inflammation, and proliferation phases, helping to stop hemorrhage, eliminate bacteria, and accelerate skin proliferation, with the growth of granulation tissue, respectively [259]. 

By adjusting the physicochemical properties of chitosan and its biological properties, with the addition of bioactives, it is possible to develop more advanced and efficient approaches for treating and controlling skin wounds. From this perspective, active compounds are incorporated into chitosan and other polymers to enhance antibacterial properties and overcome toxicity [168,260,261,262,263,264]. These antibacterial agents are biodegradable and come from renewable sources, where essential oils are one of the most promising herbal medicines to promote the wound healing process, minimizing bacterial infections [131,265,266,267,268].

## 3. Wound Healing

The skin, a multifunctional organ, protects the body from the invasion of microorganisms, provides sensory functions, and plays a crucial role in regulating body temperature [269,270,271,272,273]. However, when the skin is damaged, such as in the case of thermal burns, cuts, lacerations, surgical incisions, or chronic wounds like pressure ulcers or diabetic foot ulcers, the structure and functions of the skin can be compromised, presenting a biological burden, and psychological, social, and financial challenges for both individual patients and healthcare systems globally [274,275,276,277,278].

When wounds occur, there is susceptibility to invasion by microorganisms [279], and subsequent wound infection complicates the healing process [280]. Wound healing is a dynamic physiological process involving a multiplicity of cellular, humoral, and molecular events, aiming to restore the integrity and functionality of injured skin [281,282]. The breakdown of this complex pathway can result in a delayed or impaired healing process, resulting in acute wounds (burns, trauma) or chronic wounds (diabetes, tumor, physiological contamination) [122,283,284,285]. Wounds can further be classified according to the damaged layers of the skin, defined as (i) superficial wounds (only the epidermal layer is damaged); (ii) partial thickness wounds (damaged layers of the skin involve epidermal layers and deep layers, including blood vessels, hair follicles, and sweat glands); and (iii) full-thickness wounds (the skin down to the depth of the subcutaneous tissues is damaged) [283].

Wounds heal within 4 to 12 weeks in healthy people [223,286]. However, chronic wounds, a more severe condition, fail to make headway through the normal healing stages and are not repaired in an orderly and timely manner. Studies have shown that around 70% of chronic wounds are prone to developing an infection. These infections lead to biofilm formation, posing the threat of antibiotic resistance [258,287]. The severity of chronic wounds significantly impacts the healing process and requires careful management. 

The wound healing process can be divided into four phases: hemostasis, inflammation, proliferation maturation, and remodeling [281,286,288,289,290]. Several cellular activities occur during these phases, such as activating keratinocytes, fibroblasts, endothelial cells, macrophages, and platelets. In patients with diabetes, however, the healing process is significantly impacted due to uncontrolled blood glucose levels. This condition leads to slowed blood circulation, decreased efficiency of white blood cells, collagen synthesis, growth factor formation, inflammatory cells, fibroblasts, and proliferation and migration of keratinocytes. As a result, the formation of new tissues or vessels is delayed [291].

In the healing process, the initial response to injury is hemostasis. Clotting factors are activated and form a knot of platelets to reduce blood loss at the wound site [11,12]. The second phase includes inflammation that lasts between 24 h and 4 to 6 days. This phase begins with the emission of proteolytic enzymes and pro-inflammatory cytokines in immune cells that invade the wound area [13]. Inflammatory cells generate reactive oxygen species, and their quantity correlates with the type of wound but is generally more significant in burns and chronic wounds [292]. 

Still, in the inflammatory phase, neutrophils and macrophages remove all foreign particles and tissue debris from the wound bed, thus preventing infections. At this stage, the release of cytokines and enzymes stimulates fibroblasts and myofibroblasts, and wound exudate guarantees the moisture essential for healing [293,294,295].

The third stage is proliferation, in which platelets and leukocytes release cytokines, and stimulate angiogenesis, fibroblast proliferation, collagen, and elastin synthesis to restore the dermis, leading to scar formation [13,14]. At this stage, the intact epidermis is restored over the newly formed tissue, forming a new extracellular matrix [296]. Finally, the last stage of the healing process is remodeling, where during this phase, the newly formed capillaries regress, and most macrophages and fibroblasts undergo apoptosis [15]. Thus, the matrix composition changes, and type III collagen, initially abundant in the early stages of wound healing, is replaced by type I. This transition from type III to type I collagen increases the new tissue’s tensile strength [11,293,297,298].

Wounds are treated by debriding the infected area (eliminating dead tissue, foreign particles, and microbial biofilms), reducing pressure at the wound site, and preventing bacterial attack [299,300,301]. In addition to these procedures, a biomaterial is necessary, acting as a dressing (sterile covering) to provide the skin’s tissue regeneration characteristics and a natural barrier to the external environment, mimicking the epithelium [16,17].

## 4. Essential Oils and Biological Properties

When the skin is compromised due to injury or damage, it becomes more vulnerable to microbial infections [302]. These infections are often caused by different types of bacteria, including Gram-positive bacteria such as *Staphylococcus aureus* and *Staphylococcus epidermidis*, as well as Gram-negative bacteria like *Escherichia coli* and *Pseudomonas aeruginosa* [303,304,305]. Gram-negative bacteria have two lipid membranes and a thin layer of peptidoglycan, while Gram-positive bacteria have a single bilayer membrane surrounded by a thick layer of peptidoglycans [306,307,308,309].

*Staphylococcus aureus*, commonly found on healthy and damaged skin [310], poses a significant challenge due to its antibiotic resistance, which can impede the natural healing processes [311,312,313,314]. On the other hand, *Staphylococcus epidermidis* is beneficial in preserving the skin’s integrity and producing antimicrobial molecules that hinder the formation of biofilms by *S. aureus* [315,316,317,318]. *Escherichia coli* is known for its ability to form biofilms on various surfaces [319]. At the same time, *Pseudomonas aeruginosa* is notorious for causing persistent infections and delayed wound healing, mainly due to its high antibiotic resistance and strong biofilm-forming capacity [320,321].

Antibacterial properties are fundamental in biomedical applications, aiming to reduce inflammation caused by infections, which delay healing [24,25,26]. In this context, the emergence of resistant bacteria has spurred studies on therapeutic alternatives and the incorporation of antimicrobial agents that can enhance the efficiency of dressings [265,281,322,323]. Among these alternatives, essential oils (EOs) stand out due to their high capacity to control microbial infections [267,268,278,324,325,326]. EOs have shown promising potential in the eradication of multi-drug-resistant pathogens, as they inhibit the growth of microorganisms, creating disturbances in the cytoplasmic membrane; interrupting the proton motive force, the flow of electrons, and active transport; and hindering protein synthesis [327,328].

EOs are volatile compounds of low molar mass extracted from aromatic plants from different tissues (roots, flowers, stems, leaves, seeds, fruits, or the entire plant) [329]. They present excellent antimicrobial, antifungal, antioxidant, and anti-inflammatory properties. In addition to being biodegradable and lipophilic, they are also sedatives and analgesics with a low degree of toxicity [330,331,332,333,334,335,336]. 

Chemically, EOs are characterized by terpenes and phenylpropanoids [27,337]. Terpene compounds can be divided into two main categories: terpenes with a hydrocarbon structure (mono-, sesqui-, and diterpenes) and their oxygenated derivatives (alcohols, oxides, aldehydes, ketones, phenols, acids, esters, and lactones) [27]. The antimicrobial properties of EOs are attributed to active constituents, mainly related isoprenes, along with other hydrocarbons and phenols [338]. Therefore, the presence of phenolic compounds (carvacrol, eugenol, and thymol, among others) generates a rupture of the cytoplasmic membrane by the proton motive force, by the flow of electrons, by active transport, and also by the coagulation of cellular contents. Therefore, essential oils characterized by a high level of phenolic compounds, such as carvacrol, eugenol, and thymol, have important antibacterial activities [339], resulting in essential antibacterial properties [309,340,341,342,343,344]. Given these characteristics, between 2020 and 2023, recent studies reported using essential oils (clove, tea tree, and oregano) in wound healing [83,89,345,346].

The EO composition also influences its antioxidant potential. Phenolics and secondary metabolites with conjugated double bonds generally exhibit considerable antioxidant properties [347]. These phenolic compounds possess redox properties and are essential in neutralizing free radicals and decomposing peroxides [327]. The antioxidant activity of essential oils is also associated with some alcohols, ethers, ketones, aldehydes, and monoterpenes: linalool, 1,8-cineole, geranial/neral, citronellal, isomenthone, and menthone [348,349,350]. EOs of α-terpineol, linalool, linalyl acetate, limonene, δ-3-carene, α-pinene, and 1,8-cineole have important anti-inflammatory activity [351,352,353]. These compounds act by inhibiting histamine release or reducing the production of inflammatory mediators. Therefore, the anti-inflammatory activity of EOs can be attributed to their antioxidant activities and their interactions involving cytokines, regulatory transcription factors, and the expression of pro-inflammatory genes [341]. 

EOs can be obtained by various extraction techniques such as pressing, hydrodistillation, and steam distillation [309,341,343,354,355]. In the hydrodistillation technique, the sample is immersed in water in a distillation system with heating. The volatiles in the sample are boiled, forming a heterogeneous mixture at the end of the process. In extraction by steam distillation, water vapors pass through the sample, which entrains plant volatiles, as the plant sample is suspended and there is no direct contact with water [356,357].

The main disadvantages of applying EO are its volatility, low stability, high sensitivity [337,358], and degradation under the processing temperature [359,360,361]. Therefore, to overcome these deficiencies, EOs can be encapsulated and incorporated into polymeric matrices to increase their activity and stability, improve water solubility, and facilitate their delivery in healing applications and tissue engineering [362,363,364,365,366,367].

### 4.1. Wound Dressings

The dressing is essential for wound treatment in the medical and pharmaceutical sectors. The global market was valued at USD 12.4 billion in 2021 and is projected to grow annually by 5.3% between 2022 and 2030 [368]. The ideal dressing should promote quick healing with minimal inconvenience for the patient. Therefore, developing an advanced biomaterial for wound treatment is relevant [369,370,371]. 

The ideal wound dressing should also focus on the following characteristics: (i) provide or maintain a moist environment at the wound/dressing interface; (ii) allow gas exchange (water vapor, oxygen) between the injured tissue and the environment, aiming to maintain adequate tissue temperature, in order to improve blood flow to the wound bed; (iii) have suitable mechanical properties; (iv) provide a barrier to microorganisms; and finally, (v) remove excess exudates and toxic components from the wound surface. In addition, the dressing must be non-toxic, non-allergenic, economical, and easy to remove [248,281,289,372,373,374]. 

Potential dressings can be classified into traditional and modern dressings [281,375]. Traditional dressings, which are still widely used to treat wounds and burns, are applied to stop bleeding and prevent the wound from coming into contact with the environment [376]. They include dry dressings (gauze and bandages) and topical pharmaceutical formulations (solutions, suspensions, emulsions, creams, and ointments). Dry dressings are recommended for open, dry wounds, or secondary dressings due to their low wound coverage capacity. Topical pharmaceutical formulations, on the other hand, have the disadvantage of a short lifespan in the wound bed, especially when there is excess exudate, as they absorb fluid, lose their rheological characteristics, and become mobile [281,283]. 

While traditional dressings have limitations, modern dressings have advanced features and benefits [370]. They were developed with better biocompatibility, degradability, pain relief, and moisture retention. These modern wound dressings can be synthesized through films, membranes, scaffolds, hydrogels, nanocapsules, sponges, and nanofibers, offering a promising solution to the drawbacks of traditional dressings [273,373,377,378,379,380]. These structures comprise multilayers (absorbent and self-adhesive layers) [381,382]. The inner layers protect the wound from bacteria, cleanse infections, and heal the wound through direct contact with wound cells [383,384,385,386]. Additionally, the outer layer protects the wound from mechanical stress [387]. Incorporating essential oils is generally carried out in an external or wet layer, which provides superior healing control, eliminating contaminants such as bacteria, proteins, viruses, dyes, and/or metallic ions [388,389]. The synthesis of multilayer structures is an ideal strategy for preparing efficient multifunctional dressings [390,391]. Many studies have been carried out to investigate the potential application of EOs in wound healing through their incorporation into biomaterials such as dressings. Regarding this research, 50 studies were collected from 2010 to 2023 in the Web of Science, Scopus, Science Direct, and PubMed databases, demonstrating the synergistic use of polymeric biomaterials incorporated with EO for wound healing. According to the survey, most studies focused on clove, oregano, cinnamon, tea tree, thyme, and copaiba oil in manufacturing biomaterials such as bioactive dressings. Table 1 compiles data on essential oils and their major compounds, their biological results, systems, and the form of dressings developed.

#### 4.1.1. Films and Membranes

Films and membranes function as temporary skin substitutes, acting as a physical and mechanical barrier, mainly in managing wound infections [248,376,411]. The film can be defined as a film with restricted dimensions, that is, whose thickness is much smaller than the other dimensions [412,413]. Its production process generally involves inter- and intramolecular associations or crosslinks of polymer chains, forming a semi-rigid three-dimensional network in which the solvent is immobilized [414]. Membranes, in turn, have inherent transport properties, being a discontinuous interface between two distinct environments (permeated and retained flow). The microstructure, selectivity, and permeability of the membrane are crucial factors in the diffusion, separation, or transport of nutrients and substances. Polymeric films and membranes, therefore, tend to promote improvements in the regenerative process, including some advantages, such as the ability to absorb fluids and exudates from the wound without leaks, not requiring frequent changing and cleaning; appropriate gas exchange, providing a humid microenvironment; and wound protection against microorganisms and other toxic agents, increasing the quality of the regeneration process and reducing the risk of complications [415,416]. They also exhibit high flexibility, resistance, and transparency. Moreover, they can incorporate active ingredients, which are substances that have a therapeutic effect on the wound, such as antimicrobial agents or growth factors, and release them in a controlled or prolonged manner. This allows for a more comfortable and functional treatment, with less need to change dressings, and, consequently, reduces the dose administered, avoiding pain [417,418,419].

The main methods of processing films and membranes to obtain dense structures are the “casting” system (drying a film-forming solution, where the solvent evaporates slowly at a controlled temperature), coating, layer by layer, and extrusion, and of porous structures, they are particulate leaching, thermally induced phase separation, and electrospinning [420]. These methods allow for a wide range of film thickness, from μm to mm, depending on the methods used and the number of polymers [370].

Chitosan dressings obtained by the “casting” technique stimulate the immune system, accelerating healing due to their hemostatic properties, low immunogenicity, and high absorption. This way, they protect against microorganisms and fungi and are thermally stable [421,422]. PVA is also considered an excellent option for film/dressing production, as it has high biocompatibility, surface tension that promotes good elasticity, and good vapor transmission properties, promoting a moist microenvironment that favors epithelialization [423].

Several studies demonstrate the efficiency of dressings incorporated with clove, tea tree, and rosemary EOs due to meaningful biological interactions in the healing process [28,78,85,131,147,393,401].

Membranes with rosemary and tea tree EO incorporated into chitosan using the casting method were tested in vitro (simulating in vivo conditions through a cellular model) and in vivo (in rats) and demonstrated a significant increase in the percentage of wound contracting. Results revealed an excellent protective effect on human erythrocytes (>63%) and efficiently promoted different stages of wound healing, in addition to decreasing oxidative stress in the wound area [78,393].

The chitosan-based film incorporated with clove and tea tree essential oils was obtained by the casting method. It showed good transparency to visible light, flexibility, mechanical resistance to touch, thicknesses more minor than the dermis, and excellent wettability in distilled water and a phosphate-buffered saline solution. In in vitro tests, films obtained with 1% (*v*/*v*) of tea tree and 3% (*v*/*v*) of clove revealed activity against *Staphylococcus aureus* (halo of 6.0 mm and 9.0 mm, respectively), *Escherichia coli* (9.0 mm and 8.0 mm halo, respectively), and *Candida albicans* (7.0 mm halo in both) [28].

The dressing formulation, incorporating silver nanoparticles, sodium alginate, and essential oils of tangerine, niaouli, and clove, proved to be a prospective asset in wound care. It exhibited potential antimicrobial and antibiofilm properties, effectively treating wounds and preventing infection without antibiotics and topical antiseptic products [147]. Zein/clove EO electrospun fibrous membranes exhibited higher gas permeability with superhydrophilicity to absorb wound exudate, good biocompatibility, and antibacterial effects [131]. 

Chitosan and PVA films loaded with cinnamon and clove essential oil also showed bactericidal effects against *Staphylococcus aureus* and *Pseudomonas aeruginosa* after two hours of direct contact within the infected microenvironments [85]. Gelatin films loaded with clove essential oil and hydrotalcite nanoplates obtained by the casting method demonstrated antimicrobial activity against *Staphylococcus aureus* and *Escherichia coli* and in vitro biocompatibility. The films were non-toxic for HeLa cell lines, with cell viability above 70% [401].

The EO of *Hypericum perforatum* also demonstrated excellent potential for incorporation into different materials with healing action [122,223]. The chitosan films incorporated with *H. perforatum* oil, analyzed by the agar diffusion method, demonstrated an antimicrobial effect on *Escherichia coli* (2.9 ± 0.1 mm) and *Staphylococcus aureus* (1.97 ± 0.05 mm). Results showed that *Escherichia coli* is more sensitive to the films obtained than the bacterium *Staphylococcus aureus*. Furthermore, the films had no cytotoxic effects on NIH3T3 mouse fibroblast cells and provided a good surface for cell adhesion and proliferation [223]. 

*H. perforatum* oil incorporated into two-layer membranes formed from electrospun nanofibers of PCL and PEG exhibited controlled release and antimicrobial action against *Staphylococcus aureus* (12.9 mm) and *Escherichia coli* (11.9 mm) by the disk diffusion method. The biomaterial evaluated in vitro showed no risk of adhesion to the wound and did not demonstrate apoptotic/necrotic effects, defining it as a biocompatible material. Furthermore, the membranes had a proliferative effect on L929 fibroblast cells [122].

Lemongrass essential oil has also been studied to synthesize wound dressing materials due to its antimicrobial, antioxidant, cytotoxic, and insecticidal effects [403,404]. Chitosan films incorporated with lemongrass essential oil inhibited microbiological growth, demonstrating antimicrobial activity against *Escherichia coli* and *Staphylococcus aureus* and protection against environmental oxidative stress caused by free radicals [403]. Likewise, bioactive collagen/chitosan membranes loaded with 0.7% lemongrass essential oil showed biocompatibility and antimicrobial activity greater than 99.60%, in addition to thermal resistance to oxidation and the suppression of radicals generated by radiation gamma [404].

Other essential oils were also loaded into films and membranes with healing action [20,80,88,91,374,407]. Thyme essential oil (1.2% *v*/*v*) incorporated into chitosan generated films with antimicrobial activities against *Escherichia coli*, *Klebsiella pneumoniae*, *Pseudomonas aeruginosa*, and *Staphylococcus aureus*, mainly due to the majority constituent, the monoterpene carvacrol. The films also promoted increased water vapor and oxygen transmission rates by adding oil [374]. 

*Zataria multiflora* EO added to PVA/gelatin formed films with antioxidant and antibacterial actions against *Pseudomonas aeruginosa*. The zeta potential, particle size, and viscosity were significantly altered by adding *Zataria multiflora* to the polymer matrix [91]. Black pepper and ginger essential oils incorporated into PVA/gum arabic and chitosan generated break-resistant and flexible films with improved thermal stability, as well as significantly inhibiting the growth of *Bacillus cereus*, *Staphylococcus aureus*, *Escherichia coli*, and *Salmonella typhimurium* [80].

Loading *Ruta graveolens* essential oil into chitosan generated a film with lower permeability, water solubility, and thermal resistance, comparable to control chitosan films, without demonstrating allergic or cytotoxic reactions in erythrocytes. However, the more significant amount of EO resulted in a greater reabsorption of the implanted material in vivo in Wistar rats, with an abundant inflammatory infiltrate (typical of the healing process). This statement demonstrates that high concentrations of EO can produce greater porosity and cracking of the films and, thus, can be applied to improve cell adhesion and proliferation. However, the optimal percentage must be <1.0% [20]. 

Silica nanoparticles and patchouli essential oil incorporated into PVA/chitosan (matrix) resulted in nanocomposite films with good hygroscopicity and controlled release of the oil for more than five days. The films also exhibited a good long-term (>48 h) antibacterial effect on *Staphylococcus aureus* and low toxicity on mouse fibroblasts (L929 cells) [88]. Finally, frankincense essential oil added to gelatin/Persian gum/nanocellulose generated more stable films with constant degradation under a PBS medium. Such films showed a non-homolytic nature in vitro. Results showed that incorporating EO improved the anti-inflammatory and antibacterial activity against *Staphylococcus aureus* (standard and hospital), which can be attributed to the compound α-pinene [407].

#### 4.1.2. Hydrogels

Hydrogels are three-dimensional network structures comprising biopolymers and hydrophilic synthetic polymers with high water absorption and transport capacity [375,424,425,426,427]. Hybrid hydrogels, in turn, exploit natural polymers’ high porosity and biocompatibility and the adjustability of synthetic polymers [428,429].

Hydrogel-based wound dressings, with their promising new properties and treatment options, are poised to become leading candidates for wound treatment and repair [429]. Their unique hydration capabilities, driven by a capillary effect; the presence of carboxylic, hydroxyl, and hydrophilic compounds; and osmotic pressure [430,431], offer a refreshing and calming effect when in contact with wounds, tissues, or similar structures [69,432,433,434,435]. These properties not only make hydrogels effective as irritation-reducing agents but also equip them to prevent microbial invasion, providing a secure shield against infections while facilitating the efficient transport of bioactive molecules, such as antimicrobial agents and pharmaceuticals, even in their hydrated state [119,436,437].

Another significant advantage of hydrogels is their non-adhesive property, allowing painless dressing changes. This property enhances the patient’s comfort and promotes the healing process. Hydrogels effectively absorb wound exudates, reduce chronic infections, and promote healing without scar formation, and re-epithelialization [119,438,439]. The hydrophilic groups in the structure of hydrogel membranes enable them to be easily removed from the wound surface without breaking [345]. However, it is crucial for the resistance of hydrogel membranes to be greater than that of human skin (11.5 MPa), with the strength of this membrane primarily depending on the polymer used [440]. 

Hydrogels can be physically crosslinked through non-covalent bonds, such as ionic, hydrophobic, van der Waals, stereo complexation, and polyelectrolyte complexation, making them reversible and weak, or via chemical crosslinking through covalent bonds, or by photopolymerization (use of ultraviolet light), generating hydrogels with high stability and permanent crosslinks. Glutaraldehyde, genipin, and epichlorohydrin, among others, are some crosslinking agents used in the process [441].

Chitosan, alginate, starch, agarose, gelatin, cyclodextrin, carrageenan, fibrin, collagen, dextran, and hyaluronic acid are natural polymers that prepare hydrogels. On the other hand, we can mention synthetic polymers such as PVA, PVP, and PEG. DNA and peptides can also be used as raw materials [442]. Furthermore, in some formulations, plasticizers such as glycerol, sorbitol, and polyethylene glycol are used, which are added to increase mechanical stability and barrier capacity [443].

Nanoparticulate systems for controlled release of clove oil through a hydrogel matrix (chitosan, guar gum, and acacia gum) and PVA nanofibers demonstrated high anti-inflammatory activities in vivo. Essential oil ex vivo skin permeation results showed that nanofibers with oil nanoemulsion can sustain oil penetration through the skin [83].

Clove, oregano, and tea tree essential oils were incorporated into PVA/starch-based hydrogel membranes, resulting in good mechanical and physical properties. Furthermore, the antimicrobial activity test using the disk diffusion method revealed that the diameter of the inhibition zone was smaller against *Escherichia coli* (37.0 ± 0.29 mm) than against *Staphylococcus aureus* (39.0 ± 0.57 mm) by 0.1 mL of clove oil. The hydrogels showed high swelling capacity against water and blood, as did MgCl_2_ and NaCl solutions, as they can exude wound fluids, reducing humidity in the injured area [345]. 

The synergistic combination between PVA and chitosan functionalized with tea tree essential oil presented broad spectrums of biological action related to repairing and healing injured tissues such as burns. Results showed that the hydrogel obtained has a superhydrophilic character and a reasonable degree of swelling in the presence of fluids, distilled water, and PBS, inducing the regeneration of injured tissues [89]. Similarly, in rats, a nanoemulgel (chitosan, gelatin, and PVP/oregano essential oil) was used in vivo as a combined therapy with low-level laser therapy. Results demonstrated a maximum healing rate of 97.5%, minimal scar formation, increased granulation, enhanced re-epithelialization, and a dramatic decrease in inflammation and neutrophil infiltration within the period (days 3, 7, and 14) of treatment compared with monotherapy [346].

Other essential oils have also been loaded into a hydrogel matrix with healing action [123,398,399,400]. Innovative bioactive hydrogel dressings based on Psyllium and Carbopol supported with frankincense essential oil revealed that incorporating the oil up to 5% improves the porosity of the dressing, increases the water vapor transmission rate, and has antioxidant activity. The dressing attenuated the microbial growth of *Staphylococcus aureus*, *Escherichia coli*, and *Candida albicans* after 18 h and presented faster healing in vivo, with better biochemical parameters [398].

The nanogel obtained from the encapsulation of *Satureja khuzistanica jamzad* essential oil in chitosan nanoparticles promoted an increase in the thermal stability of the oil. The product demonstrated antibacterial effects against Gram-positive and Gram-negative bacteria (MIC between 7.8 and 500 mg/mL) and also acceptable anticancer activities against the KB and A549 tumor cell lines (IC50 of 5.59 µg/mL and 7.78 µg/mL, respectively) [399].

The antibacterial hydrogel obtained based on polysaccharides (carboxymethyl chitosan and carbomer 940) associated with essential oils (eucalyptus, ginger, and cumin) was applied to repair burned skin. The hydrogels incorporated with eucalyptus oil exhibited high antibacterial activity against *Staphylococcus aureus* (inhibition rate of 46.26 ± 2.22%) and *Escherichia coli* (inhibition rate of 63.05 ± 0.99%), along with cell viability of 92.37%, by the MTT assay using L929 cells. Results also showed that this hydrogel induced a significant migration of L929 cells (59.83% after 24 h and 100% after 48 h) and accelerated wound healing in vivo, promoting recovery of the dermis and epidermis [400]. Thyme essential oil incorporated (15%) into κ-carrageenan and PEG hydrogel membranes also showed in vitro antimicrobial activity (>95%) against *S. aureus* and *E. coli* with a slow-release profile. Cytocompatibility, determined with HEK293 cells (human embryonic kidney cell line 293) using the MTT assay, demonstrated that the membranes exhibited ~89% cell viability [123].

#### 4.1.3. Nanofibers

Nanofibers, with their unique properties of a large specific surface area, high porosity, excellent pore interconnectivity, and good mechanical properties [75,444,445], offer distinct advantages for wound healing. These properties aid in cell adhesion and proliferation, facilitate permeability to moisture and gasses, and promote cell growth. They also allow the absorption of additional exudates containing nutrients for bacterial growth [446,447,448,449,450,451,452]. This unique combination of properties makes nanofibers promising candidates for dressing materials, providing an ideal environment for wound healing. Among the techniques used to obtain nanofibers, electrospinning is a simple, efficient, and economical method to produce nanoscale fibers by applying an electric field to polymer-based solutions [453,454,455]. The diameter of the nanofibers obtained by this technology can reach several hundred nanometers or even less than one hundred nanometers [456]. The internal pore structure of electrospun nanofibers can be controlled, as well as their composition and structure [457,458]. During the electrospinning process, fiber morphology is affected by multiple factors, such as polymer properties, electrospinning process parameters, and environmental parameters, which regulate electrospun fibers’ shape, diameter, and quality [19].

The high porosity of nanofibers provides a contact surface more suitable for gas exchange and liquid absorption, which generates excellent permeability. This, in turn, keeps the wound moist and acts as a strong barrier against microbial invasion, ensuring the safety of the wound [459,460,461,462]. Simultaneously, the high flexibility of the nanofiber makes the dressing suitable for different parts and shapes of the wound [463,464].

The polymeric materials used to synthesize nanofibrous dressings include natural and synthetic polymers. Natural polymers easily form strong hydrogen bonds with the aqueous solution, not allowing spinning, as they form a highly viscous solution [456]. To improve spinnability, chitosan, for example, is usually mixed with other polymers, such as PVA, PLA, PEO, and PCL, to obtain nanofibers for wound dressings [24,160,465,466,467,468,469,470].

Solution blow spinning can produce PLA and PVP nanofibers loaded with copaiba oil (*Copaifera* sp.). Adding PVP to PLA enabled a higher rate of controlled oil release in vitro. The nanofibers obtained from blends containing more significant amounts of PVP revealed greater antimicrobial action against *Staphylococcus aureus* [392].

*Zataria multiflora* EO was incorporated into electrospun nanofibers based on chitosan/PVA/gelatin. The dressing generated demonstrated the total inhibition of the growth of *Staphylococcus aureus*, *Pseudomonas aeruginosa*, and *Candida albicans* after 24 h of incubation at a concentration of 10% EO. In vitro biocompatibility tests on mouse fibroblast cells (L929) have considered the product as non-toxic [79]. 

The electrospun nanofibers based on concentrated collagen hydrolysate loaded with oregano and thyme essential oils showed antimicrobial properties against *Staphylococcus aureus*, *Escherichia coli*, *Pseudomonas aeruginosa*, and *Candida albicans*. They do not present cytotoxicity in in vitro cultivation tests with NCTC 929 clones of fibroblast cells (oil concentrations below 500 μg/mL) [395]. 

PLCL and silk fibroin, also loaded with oregano essential oil, produced biocompatible electrospun nanofibers active against Gram-positive and Gram-negative bacteria. By a histological analysis, the incorporation of 5% of the oil improved the quality of wound healing, indicating neoepithelialization, granulation tissue formation, angiogenesis, and collagen deposition [396]. Likewise, materials obtained by electrospun PLCL nanofibers loaded with ZnO nanoparticles and oregano essential oil revealed intense antibacterial and antioxidant activities in vitro. In wound healing in vivo, bioactive membranes acted in epithelialization, granulation tissue formation, and angiogenesis. In the inflammatory cycle, membranes showed an anti-inflammatory effect by downregulating pro-inflammatory cytokines [402].

The PLGA/gelatin nanofibrous dressings added with bioactive glass and oregano essential oil showed rapid hemostasis, improved chemotactic response, more significant angiogenesis, decreased bacterial colonization, and anti-inflammatory response in vitro. Preliminary biocompatibility testing in a subcutaneous implantation model revealed membrane arrangement with endogenous cellular components and neo-tissue formation, promoting membrane remodeling in vivo. In in vivo tests, the synthesized dressings substantially improved wound re-epithelialization and neo-vessel formation, inducing macrophage polarization, suppressing inflammation, and promoting scar-free healing [409]. 

The sodium alginate solution, PVA, and essential oils (cinnamon, clove, and lavender) generated electrospun nanofibers with good antibacterial properties against *Staphylococcus aureus*, which makes them a suitable replacement for antibiotics. Nanofibers loaded with cinnamon oil showed better antibacterial properties compared to other oils. Results demonstrated that cotton gauze coated with nanofibers showed more excellent liquid absorption than plain cotton gauze [81]. Additionally, PCL/gelatin and PCL nanofibers containing clove essential oil and peppermint essential oil, respectively, produced by electrospinning, showed antibacterial activity against *Staphylococcus aureus* and *Escherichia coli* and had no cytotoxic effects on normal human dermal fibroblast cells [98,394]. 

The electrospun nanofibers formed from PU loaded with lavender oil and silver nanoparticles showed improved hydrophilicity, guaranteed fibroblast proliferation in their natural form, and excellent bactericidal properties against *E. coli* and *S. aureus* [111]. Clove oil encapsulated with chitosan/PEO under the electrospinning technique generated nanofibers with good antibacterial activity against *Staphylococcus aureus* and *Escherichia coli* and non-cytotoxic behavior against human fibroblast cell lines, with good wound healing potential [130]. In the study with combinations of polymers (hyaluronic acid/PVA/PEO) and antimicrobial agents (cinnamon oil and zinc oxide nanoparticles), it was observed that electrospun nanofibers inhibited the growth of *Staphylococcus aureus* in vivo, being an innovative trend to avoid the use of an antibiotic [86].

Lemon balm and dill essential oils encapsulated in collagen/chitosan hydrolysates generated electrospun nanofibers with good in vivo biocompatibility and improved antimicrobial activity against *Staphylococcus aureus*, *Enterococcus faecalis*, *Candida albicans*, and *Candida glabrata* [359]. Black pepper essential oil encapsulated in PLA coated with chitosan demonstrated that electrospun porous biocompatible fibers have a more significant antimicrobial effect than uncoated fibers. Chitosan coating stopped bacterial growth and improved hydrophilicity, promoting cell adhesion and proliferation [282]. *Zingiber cassumunar Roxb* essential oil (Plai) incorporated into a polymeric mixture (PLA and PEO) showed good antibacterial properties against *S. aureus* and *E. coli* and was not toxic to human fibroblasts and keratinocyte cells [132].

#### 4.1.4. Scaffolds

Scaffolds are made of biomaterials and have a three-dimensional structure that supports and promotes cell development, an essential step in tissue regeneration [2,471,472]. Furthermore, they serve as cell carriers and provide a growth environment for cellular communication and functional maintenance [473,474,475,476,477]. Scaffolds must simulate the cellular microenvironment in vivo and maintain cell viability and function, aiding cell proliferation, differentiation, and biosynthesis [478,479,480,481].

The chemical structure of the source biomaterial and its processing determine the functional properties and the interaction of cells with the scaffold [482]. Therefore, scaffolds can be designed in two ways: porous or dense. Porosity is a fundamental characteristic of adequate cell housing. It provides scaffold–cell interaction (microporosity) and optimizes the transport of nutrients and gasses through the three-dimensional matrix (macroporosity) and its vascularization. Porosity should not be excessive to not compromise the scaffolds’ mechanical stability [483,484,485,486]. In addition, the scaffold must be biocompatible, biodegradable, and non-toxic [2,450,478,487,488,489].

Scaffolds can be synthesized from natural polymeric materials (e.g., collagen, alginate, chitosan, decellularized matrices) or synthetic materials (e.g., PEG, PU, PLA, PCL, and poly(ethylene glycol) diacrylate (PEGDA)), intended to replicate the natural three-dimensional (3D) environment (extracellular matrix (ECM)) so that cells can proliferate and organize into tissues or organs while maintaining their specialized configurations and morphologies [490,491,492,493,494,495,496,497,498,499,500,501,502]. Furthermore, they have significant clinical relevance for repairing or regenerating diseased or damaged tissues [479], and adding essential oils to their structure tends to overcome biocompatibility and biological activity challenges.

*Satureja mutica* and *Oliveira decumbens* essential oils were encapsulated in the core of nanofibrous scaffolds synthesized based on chitosan/PVA and PVP/maltodextrin (core and shell, respectively). The addition of such essential oils increased the antioxidant and antimicrobial activity of the scaffolds, completely inhibiting the growth of standard strains of *Pseudomonas aeruginosa*, *Escherichia coli*, *Staphylococcus aureus*, *Candida dubliniensis*, and *Candida albicans*. The wide range of antimicrobial effects protects the wound site from probable infection. It is related to its phenolic compounds (carvacrol and thymol) as they disturb the cell cytoplasm’s balance of ions and pH [84].

The encapsulation of oregano essential oil in PVDF through electrospinning demonstrated, by an in vitro analysis, that the obtained scaffold was biocompatible in normal human cells and had significant antioxidant and antitumor activities against the liver cancer cell line (Huh7) and triple-negative breast cancer (MDA-MB 231), even after six months of storage at room temperature [406].

The antibacterial properties of cinnamon essential oil and eugenol combined with PCL have also been studied for medical applications. The highly porous scaffolds with 30% eugenol and cinnamon oil showed a small inhibition halo for Gram-positive microorganisms (between 21.07 ± 0.30 and 28.63 ± 0.17 mm). Adhesion experiments, on the other hand, revealed a significant decrease in adherent and planktonic bacteria recorded for *Staphylococcus aureus*, *S. epidermidis*, and *Escherichia coli*, showing an anti-adhesive characteristic. Results also confirmed the absence of a cytotoxic effect in an in vitro assay with the human osteosarcoma cell line Saos-2 [93].

Cellulose acetate was also electrospun to encapsulate retinyl palmitate and clove essential oil to obtain a wound dressing. The effect of incorporating clove oil on the scaffolds’ antioxidant activity, antibacterial activity, cell viability, and release behavior was investigated. The results showed that the 5% *w*/*w* ratio of the oil resulted in a biocompatible scaffold with L929 fibroblast cells, in addition to antioxidant and antibacterial properties against *E. coli* and *S. aureus* [410]. 

Another technique to obtain scaffolds with a macroporous profile is the “cryogelation” technology (gelation at cryogenic or freezing temperatures) [503]. Cryogels are synthesized at subzero temperatures where most of the solvent phase is frozen while a small portion remains in the liquid phase, called the thawed liquid microphase. Dissolved solutes concentrate and can undergo necessary chemical reactions, leading to the formation of cryogels. Ice crystals are porogenic elements that leave behind interconnected pores when thawing [478,504,505]. Thus, some parameters must control the physical properties of these materials, such as the degree and type of crosslinking, composition, temperature and incubation time, and freezing rate [506]. 

Scaffolds manufactured by cryogelation are soft, spongy, highly porous, physically stable, and elastic, and can come in various shapes [505]. Chitosan cryogel scaffolds loaded with *Hypericum perforatum* oil demonstrated excellent antimicrobial effects against *E. coli* and *L. pneumophila* and can be applied in tissue engineering as dressings for exuding and long-term healing wounds [300].

Similarly, gelatin and chitosan cryogels with incorporated clove oil were macroporous, were biodegradable, had mechanical properties similar to commercial skin substitutes, were cytocompatible, were antibacterial, and allowed prolonged oil release for up to at least 14 days. The results also showed that clove essential oil, being antimicrobial, is a scaffold formulator that acts to close scratch wounds more quickly in vitro, improving the migration of fibroblasts [405]. 

Aerogels are also functional materials of fiber and air scaffolds without water or any other solvent. They have been prepared from various organic and inorganic materials and used in numerous medical and non-medical applications [507,508]. Cinnamon essential oil and chitosan incorporated into cellulose nanofiber bioaerogel were obtained by high-pressure homogenization and freeze-drying. Results showed improved mechanical properties and in vitro stability in water for more than four weeks at room temperature. They also demonstrated better biocompatibility, intense antibacterial activity against *S. aureus* and *E. coli*, and the proliferation of L929 fibroblast cells, and they can be used as surgical sutures and in tissue regeneration [408]. 

#### 4.1.5. Other Modern Wound Dressings

Polymeric nanocapsules hold immense potential as healing materials. They are vesicular nanostructures comprising an oily core enveloped by a polymeric wall. Their unique properties, such as controlled drug release, high encapsulation of lipophilic components, and drug protection [365,509,510,511], pave the way for promising advancements in wound healing and antibacterial treatments.

The effectiveness of chitosan films, when incorporated with copaiba oil nanocapsules, is undeniable. These films have demonstrated potent in vitro antibacterial effects against *Staphylococcus aureus* and *Pseudomonas aeruginosa*. This antibacterial activity is likely attributed to the presence of lipophilic terpenes (β-caryophyllene), which can disrupt the bacterial cell’s integrity, leading to the leakage of intracellular contents and subsequent cell death. The presence of copaiba oil nanocapsules in the films also enhances their homogeneity and surface characteristics [257], further bolstering their efficacy.

Matrices composed of chitosan-based emulsions functionalized with cabreuva essential oil (*Myrocarpus fastigiatus*) and PVA were also synthesized to obtain smooth, flexible, and thin dressings capable of acting as a delivery vehicle. The dressings were effective against *S. aureus* and *S. epidermidis*, promoting cell regeneration after 24 h of contact and the absence of cytotoxicity in HaCaT cells [82].

Sponges are also potential dressings for treating wounds with high exudate [512,513]. In addition to their good ability to absorb wound exudates, sponges are also suitable for treating bleeding wounds. Therefore, developing porous sponges with hemostatic, antibacterial, and antioxidant performance is advantageous due to their distinct three-dimensional structures, which allow them mechanical stability. It is worth highlighting that the three-dimensional structure strengthens the adhesion and proliferation of cells recruited for wound healing [514].

The synergistic effect of the PVA-based sponge compound, marjoram essential oil, and kaolin effectively controlled hemorrhage due to the great water absorption. Results demonstrate excellent antibacterial performance against *Escherichia coli* and *Bacillus cereus*, and good antioxidant properties and thrombogenicity, developing high thrombus mass and hemocompatibility, in addition to its notable safety regarding fibroblast cells, making it promising for wound healing [87].

Another type of modern dressing is nanocomposites, which can be formed from clay and a polymer. The presence of clay prevents the evaporation of the antimicrobial essential oil as it enters the clay galleries due to interaction with the organomodified clay. The ability of tea tree essential oil to eliminate resistant microorganisms in new bionanocomposites, with montmorillonite clay and PLA, was analyzed. The results showed the effectiveness of tea tree oil against *Escherichia coli* and *Staphylococcus aureus* bacteria [397].

## 5. Materials and Methods

**Figure 2:**
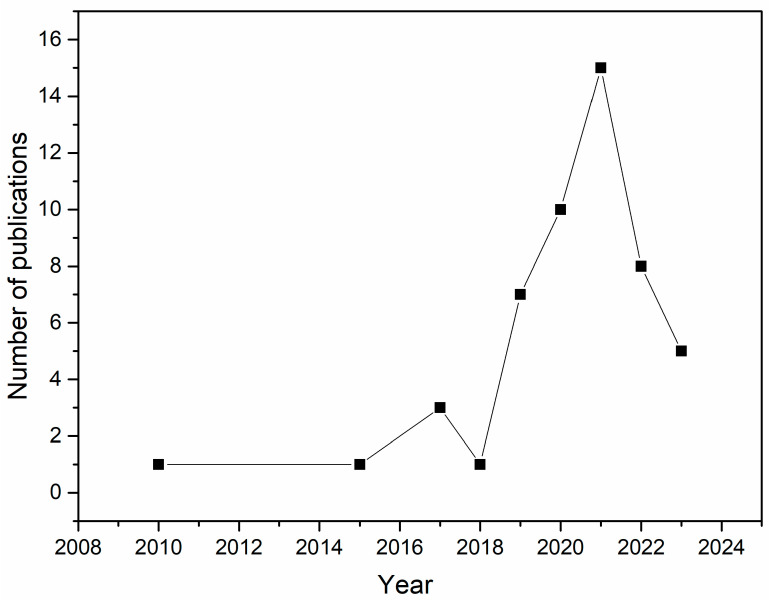
A graphical representation of the number of publications on EOs with antimicrobial and anti-inflammatory activity in the past 13 years.

The present study was based on scientific publications on EOs from plants for dressing materials with antimicrobial and anti-inflammatory activity between 2010 and 2023. Figure 2 emphasizes the gradual growth in publications on this topic from 2019 to 2021. As a result, the production of this review highlighted aromatic plants with antimicrobial and anti-inflammatory activity, as well as the main chemical components of EOs, systems, and forms of dressings and their biological results, which are listed in Table 1.

### 5.1. Search Strategy and Inclusion and Exclusion Criteria

**Figure 3:**
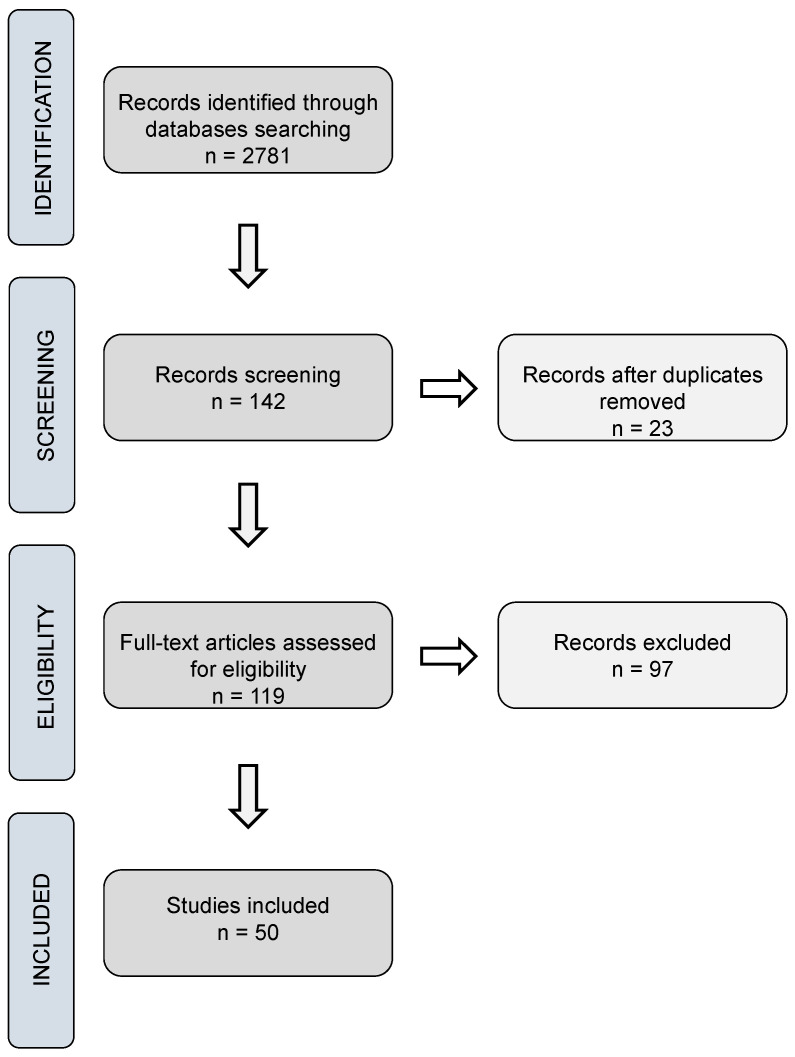
Search strategy flowchart.

The search for information on the chemical composition of EOs and tests performed was implemented considering all articles published in the past thirteen years (2010–2023) in the literature databases Web of Science (https://www.webofscience.com, accessed on 17 February 2023), Scopus (https://www.scopus.com, accessed on 17 February 2023), Science Direct (https://www.sciencedirect.com/, accessed on 15 February 2023), and PubMed (https://pubmed.ncbi.nlm.nih.gov/, accessed on 17 February 2023). The primary keyword “essential oil”, “tissue engineering”, “wound healing”, “antimicrobial”, “anti-inflammatory”, and “polymers” activities were searched for and combined in the titles and abstracts. Inclusion criteria for sections of this study were accessed for systems (polymers—essential oil) and assays to support antimicrobial and anti-inflammatory activity. Searches performed with review articles, books, book chapters, and non-medical applications were excluded. Figure 3 summarizes the general methodology, highlighting the articles collected in each database, duplicated, and excluded, and finally, how many were selected for writing this review.

### 5.2. Study Records: Data Management

Table 1 describes the mechanism used for data management, uniformly naming the plant species, main components, and antimicrobial and anti-inflammatory effects of the EO.

## 6. Conclusions and Future Perspectives

The understanding that the application of polymers with biocompatible and biodegradable characteristics, such as chitosan, can offer practical alternatives for the treatment of severe wounds and burns, as it presents excellent biocompatibility, biodegradability, and non-toxicity, has been proven through numerous studies. Ideally, the scaffold/membrane/film/hydrogel material should induce as little pain as possible, allow rapid healing, and direct the growth in defect-free epidermal cells. To this end, various bioactive compounds have also been incorporated into dressings to provide these desired characteristics. EOs act as secondary metabolites, defending the host from microbial invasion. The antimicrobial effect comes mainly from known polyphenols and terpenes: monoterpenes, eugenol, cinnamaldehyde, carvacrol, and thymol.

Many plant species that contain EOs with antioxidant, anti-inflammatory, and antimicrobial activities widely studied have shown promise for studies in the biomedical area; among them are *Syzygium aromaticum* or *Eugenia caryophyllata* (clove), *Organum vulgare* (oregano), and *Cinnamomum zeylanicum* (cinnamon). These plants are good candidates for clinical trials due to their proven effects on wound healing and reducing the incidence of inflammatory cells at the wound site. They are also of low toxicity and are widely available and used in the food and cosmetics industry.

Studies with polymeric membranes loaded with essential oils on the formation of new blood vessels in the wound area, formation of epithelial tissue, and evaluation of the reduction in oxidative stress are also approaches that should be considered in the future. These approaches aim to improve the local oxygenation process of the affected area and reduce scars and tissue regeneration. Furthermore, these materials can be suitable substitutes for conventional antibiotics due to their potent antimicrobial activities against various pathogens.

The integration of engineering techniques, such as electrospinning, rotospinning, and 3D bioprinting, could also represent, shortly, a positive impact on the generation of hybrid materials from polymers and EOs by integrating cells, growth factors, and biomaterials with personalized structures, mimicking natural characteristics of organs and tissues. From this perspective, the continued need for research into EOs for bioactives is clear, valuing traditional knowledge and biodiversity. Therefore, this review offers an overview of the evidence found in the past thirteen years of using EOs in wound healing, as well as the dressing form and biological outcomes.

## Figures and Tables

**Table 1:** Summary of the primary information and results found in the studies of EO that have shown curative activity in the past thirteen years.

Publication Year/Species /Essential Oil	Major Compounds	Wound Dressing System	Form	Biological Results	Refs.
2010 *Thymus vulgaris* Thyme	carvacrol and thymol ^a^	CS (low molecular weight, degree of deacetylation ~85)	Films	Antibacterial activities and antioxidants	[374]
2015 *Copaifera* Copaiba	β-cubebene, β-, α-caryophyllene, α-bergamotene, β-, δ-cadinene, β-, α-selinene, β-, α-bisabolene	PLA (Mw~66,000 g/mol) and PVP (Mw~55, 000 g/mol)	Nanofibers	In vitro release tests of oil volatiles demonstrated a higher release rate and had greater antimicrobial action against *Staphylococcus aureus* in fibers containing PVP	[392]
2017 *Hypericum perforatum*	chemical composition not reported	CS (low molecular weight, degree of deacetylation of 85%)	Films	The films had antimicrobial activity against the activity of *Escherichia coli* and *Staphylococcus aureus*; they had no cytotoxic effects on NIH3T3 fibroblast cells and provided a good surface for cell attachment and proliferation	[223]
2017 *Rosmarinus officinalis* Rosemary	1,8-cineole, α-pinene, camphor, and camphene ^a^	CS (deacetylation degree of 34% and molecular weight of 128 kg/mol)	Membrane	They showed high percentages of inhibition of erythrocyte hemolysis (>63%) and relatively low antioxidant capacity by the ABTS radical (≈6 to 9%)	[393]
2017 *Melaleuca alternifolia* Tea tree	terpinen-4-ol, γ-terpinene, α-terpinene ^a^				
2017 *Zataria multiflora*	thymol, carvacrol, p-cymene, and γ-terpinene ^a^	PVA (60–70 kDa)/gelatin (type A, 50–100 kDa)	Films	Considerably increased the antioxidant and antibacterial activities of the dispersions. *Pseudomonas aeruginosa* was the most resistant bacteria	[91]
2018 Cinnamon	cinnamaldehyde, cinnamyl acetate, caryophyllene, linalool, and eugenol ^a^	Sodium alginate/PVA (90% hydrolyzed having Mw of 70,000)	Nanofiber	Good antibacterial properties against *Staphylococcus aureus*	[81]
2018 Clove	eugenol, eugenyl acetate, β-caryophyllene ^a^				
2018 Lavender	linalyl acetate, linalool, lavandulyl acetate, camphor, 1,8-cineole ^a^				
2019 *Melaleuca alternifólia* Tea tree	terpinen-4-ol (45.23%), γ-terpinene (23.07%), α-terpinene (10.84%)	CS (highly viscous)/PVA (molecular weight ~31,000)/glycerol	Films	Significant increase in wound contraction percentage; decreased oxidative stress in the wound area; re-epithelialization associated with activated hair follicles	[78]
2019 *Rosmarinus officinalis* L. Rosemary	1,8-cineole (53.67%), α-pinene (13.94%), camphor (10.43%)				
2019 *Eugenia caryophyllata or Syzygium aromaticum* Clove	eugenol, eugenol acetate, and β-caryophyllene ^a^	CS (shrimp shells, medium molecular weight: 230–250 kg/mol; degree of deacetylation: 85%)	Films	Inhibition against *Staphylococcus aureus*, *Escherichia coli*, and *Candida albicans*	[28]
2019 *Melaleuca alternifolia* Melaleuca	composition and % not reported				
2019 *Eugenia caryophyllata* Clove	eugenol (78.00%), β-caryophyllene (13.00%)	PCL (Mw = 80 kg/mol)/gelatin (~300 g Bloom, type A)	Nanofiber Mats	Did not have cytotoxic effects on normal human dermal fibroblast (NHDF) cells; exhibited antibacterial activity against *Staphylococcus aureus* and *Escherichia coli*	[98]
2019 *Mentha piperita* Peppermint	menthol ^a^	PCL (Mw = 80,000)	Nanofiber Mats	Exhibited antibacterial activity against *Staphylococcus aureus* and *Escherichia coli*; did not have cytotoxic effects on normal human dermal fibroblast (NHDF) cells	[394]
2019 *Zataria multiflora*	thymol (52.80%), o-cymene (13.89%), carvacrol (5.97%)	CS (Mw = 600–800 kDa)/PVA (Mw = 72 kDa)/gelatin (edible bovine)	Nanofiber	Completely inhibited the growth of *Staphylococcus aureus*, *Pseudomonas aeruginosa*, and *Candida albicans* after 24 h of incubation; exhibited no cytotoxicity for L929 cells and showed suitable biocompatibility	[79]
2019 *Lavandula angustifolia* Lavender	linalool, terpene-4-ol, linalyl acetate, camphor, β-caryophyllene, and lavandulyl acetate ^a^	PU (Mw = 110,000)/silver nitrate (AgNO_3_, purity ≥ 99.0%)	Nanofiber	Offer protection against external agents (*E. coli* and *S. aureus*); promote the regeneration of new tissue	[111]
2019 *Hypericum perforatum*	chemical composition not reported	PEG (molecular weight of 10,000 g/mol)/PCL (molecular weight of 80,000 g/mol)	Membrane	Membranes exhibit antimicrobial activity against *Staphylococcus aureus* and *Escherichia coli*; do not have the risk of adhesion to wound; do not have apoptotic/necrotic effects, being biocompatible; and have proliferative effect on cells	[122]
2020 *Zingiber officinale Roscoe* Ginger	α-zingiberene (29.21%), β-cedrene (19.94%), α-curcumene (13.88%), β-bisbolene (11.54%), β-sesquiphellandrene (8.40%)	PVA/gum arabic/CS (degree of deacetylation of 90%)	Films	The films significantly inhibited the growth of *Bacillus cereus*, *Staphylococcus aureus*, *Escherichia coli*, and *Salmonella typhimurium*	[80]
2020 *Piper nigrum* Black pepper	caryophyllene (28.42%), followed by 3-carene (6.73%) and D-limonene (6.13%)				
2020 *Hypericum perforatum*	chemical composition not reported	CS (low molecular weight)	Cryogels	Exhibited excellent antimicrobial activity against *E. coli* and *L. pneumophila* and antioxidant effects	[300]
2020 *Thymus vulgaris* Thyme	carvacrol (57.40%), α-terpinene (32.40%), o-cymol (3.90%)	Collagen hydrolysate (bovine pelt)	Nanofibers	Antimicrobial properties against *Staphylococcus aureus*, *Escherichia coli*, *Pseudomonas aeruginosa*, and *Candida albicans*; non-cytotoxic and biocompatible, and properties of antioxidants	[395]
2020 *Origanum vulgare* Oregano	thymol (64.40%), carvacrol (27.60%)				
2020 *Myrocarpus fastigiatus* Cabreuva	*E*-nerolidol ^a^	PVA (hydrolysis degree of 98% and a molecular weight in the range of 78 kg/mol)/CS (deacetylation degree of 85% and molecular weight of 3.2 × 102 kg/mol)	Nanocapsules’ Film	Effectiveness against microorganisms such as *S. aureus* and *S. epidermidis*, capacity to produce cell regeneration after 24 h of contact time, and no cytotoxicity in HaCaT cells	[82]
2020 *Syzygium aromaticum* Clove	eugenol (88.85%)	PVA (molecular weight of 146–186 Kg/mol; degree of hydrolysis of 98.0–98.8 moL %)/CS (low MW of 1526.454 g/mol; deacetylation degree of 90–95%)	Nanoemulgel and Nanofibers	Superlative anti-inflammatory activity against croton oil-induced mouse skin inflammation model; presents cutaneous safety profile	[83]
2020 *Ruta graveolens*	2-nonanone (23.50%) and 2-undecanone (42.60%)	CS (from shrimp shells, molecular weight: 144.000, deacetylation degree: 89–90%)	Films	Good resorptions of the films with abundant inflammatory infiltrate; no allergic or cytotoxic reactions in erythrocytes were present	[20]
2020 Mandarin	composition and % not reported	Silver nanoparticles/sodium alginate	Films	The niaouli EO is more efficient against microbial attachment and biofilm formation, while mandarin and clove EOs are more efficient at diminishing microbial growth in planktonic, free-floating cells	[147]
2020 Niaouli					
2020 Clove					
2020 *Eugenia caryophyllata* Clove	eugenol ^a^	Zein (from corn)/PEO (Mv of 100,000)	Films	Exhibited good gas permeability to allow gas exchange; showed superhydrophilicity to absorb the wound exudate and good biocompatibility and antibacterial effects	[131]
2020 *Origanum vulgare* Oregano	carvacrol ^a^	PLCL/cocoons of Bombyx mori silkworm	Nanofiber	It has turned out to be biocompatible, anti-adhesive, and antibacterial against both Gram-positive and Gram-negative bacteria; accelerated wound contraction with complete epithelialization, collagen deposition, and angiogenesis	[396]
2020 *Melaleuca alternifolia* Tea tree	terpinen-4-ol ^a^	PLA/montmorillonite clay (Cloisite20A)	Bionanocomposites	Showed antibacterial test against *Escherichia coli* and *Staphylococcus aureus* bacteria present in a wound environment	[397]
2021 Frankincense	chemical composition not reported	Psyllium/Carbopol 940	Hydrogel	They showed antioxidant efficiency, excellent barrier potency against external microorganism attacks, and efficient antimicrobial activities against *S. aureus*, *E. coli*, and *C. albicans*; induced faster wound healing with improved biochemical parameters compared with oil-free hydrogel	[398]
2021 *Satureja khuzistanica jamzad*	carvacrol (98.18%)	CS (data not mentioned)	Nanogel	Showed antimicrobial properties appearing not only on the Gram-positive bacteria but also on the majority of Gram-negative bacteria; its anti-tumor effect was noticeable on KB-cell line	[399]
2021 *Melissa officinalis* Lemon balm	citronellal (13.70%), citral (geranial and neral, 9.90%), and β-caryophyllene (4.60%)	Collagen hydrolysate (bovine skin and rabbit collagen glue)/CS (highly viscose in the form of crystals, viscosity of 1267 Mpa.s)	Nanofibers	Improved the antimicrobial activity against *Staphylococcus aureus*, *Enterococcus faecalis*, *Candida albicans*, and *Candida glabrata*; showed good biocompatibility	[359]
*Anethum graveolens*	o-cimol (30.71%) and α-phellandrene (23.21%)				
2021 *Piper nigrum* Black pepper	limonene ^a^	PLA (MW = 120,000 g/mol)/CS (medium molecular weight)	Fibers	Improved the hydrophilicity of the fibrous mats, enhanced EO’s antibacterial potential, and promoted cell adhesion and proliferation	[282]
2021 *Satureja mutica*	carvacrol (64.04%), ρ-cymene (12.11%), γ-terpinene (6.22%)	PVP (Mw of 360000)/PVA (Mw of 72000)/CS (molecular weight: 50–190 kg/mol, deacetylation degree: 75–85%)	Scaffolds	Enhanced the antioxidant activity of the scaffolds and broadened the microbicidal activity	[84]
2021 *Oliveria decumbens*	γ-terpinene (25.87%), thymol (20.32%), carvacrol (18.77%), ρ-cymene (12.72%), myristicin (9.89%), and limonene (5.5%)				
2021 *Cinnamomum zeylanicum* Cinnamon leaf	eugenol (79.00%)	CS (Mw = 100–300 kg/mol)/PVA (Mw = 72 kDa, 88% hydrolyzed)	Films	It has the potential to increase the antimicrobial activity against *Staphylococcus aureus* and *Pseudomonas aeruginosa*	[85]
2021 *Eugenia caryophyllus* Clove	eugenol (81.00%)				
2021 *Syzygium aromaticum* Clove	eugenol (71.43%), β-caryophyllene (10.32%), and eugenol acetate (8.32%)	CS (not mentioned information)/PEO (Mw of 600 KD)	Nanofibers	Showed no cytotoxicity against fibroblast cell lines and showed effective antibacterial activity against *Staphylococcus aureus* and *Escherichia coli*, and wound healing activity	[130]
2021 *Syzygium aromaticum* Clove	eugenol, eugenyl acetate, and caryophyllene ^a^	PVA (degree of polymerization = 1500)/starch	Hydrogel Membrane	The antibacterial efficacy was inspected against *Escherichia coli* and *Staphylococcus aureus* and provided a moist environment by meaningfully reducing the transmission of moisture from the wound bed	[345]
2021 *Melaleuca alternifolia* Tree tea	alpha, γ-terpinen-4-ol, cymene, and cineole ^a^				
2021 *Origanum vulgare* Oregano	carvacrol, β-fenchyl alcohol, thymol, and γ-terpinene ^a^				
2021 Eucalyptus	eucalyptol (83.27%), D-limonene (5.82%), o-cymene (3.46%)	CMC (molecular weight concentrated at 195.7 kg/mol and 2.0 kg/mol; the degree of substitution was 73.73%)/CBM 940 (molecular weight concentrated at 1894.7 kg/mol and 15.8 kg/mol)	Hydrogel	Show high antibacterial activity and cell migration activity and a significant effect on skin repair in vitro and in vivo	[400]
2021 Ginger	1,3-cyclohexadiene (39.81%), cyclohexene (14.92%), β-bisabolene (9.81%), benzene (7.18%), and γ-muurolene (7.15%)				
2021 Cumin	anethole (74.53%) and α-pinene (15.48%)				
2021 *Cinnamomum verum* (*zeylanicum*) Cinnamon	eugenol and cinnamaldehyde ^a^	Hyaluronic acid (Mw of 1000 kg/mol)/PVA (Mw of 75 kg/mol, 98% hydrolysis)/PEO (≥95%, Mw of 900 kg/mol)/zinc acetate dihydrate (Mw of 183.48, 99.99%)	Nanofiber	Showed good physicochemical properties, cytocompatibility, antibacterial activity, and enhanced healing of *S. aureus*-inoculated full-thickness incision wounds in a rat model	[86]
2021 *Syzygium aromaticum* Clove	eugenol (84.10%)	Bovine gelatin/hydrotalcite	Films	Showed antimicrobial activity against *Staphylococcus aureus* and *Escherichia coli*; good in vitro biocompatibility and were non-toxic	[401]
2021 *Origanum majorana* Marjoram	terpinen-4-ol, (+)-*cis*-sabinene hydrate, γ-terpinene and terpinolene, thymol, and carvacrol ^a^	PVA (Mw = 72 kg/mol)/kaolin (hydrated aluminum silicate)	Sponges	Exerted exceptional antibacterial performance against *Escherichia coli* and *Bacillus cereus*, along with remarkable antioxidant properties; demonstrated significant thrombogenicity, developing high thrombus mass and hemocompatibility; and remarkable safety toward fibroblast cells	[87]
2021 *Pogostemon cablin* Patchouli	patchouli alcohol (43.78%), phthalene (16.03%), δ-guaiene (11.86%), γ-patchoulene (7.59%), and α-guaiene (4.69%)	CS (viscosity > 400 mPa.s)/PVA (alcoholysis degree: 99.0–99.4 mol%, viscosity: 12.0–16.0 mPa.s)	Film Nanocomposite	Exhibited good long-term (>48 h) antibacterial effect on *Staphylococcus aureus* and non-toxicity on mouse fibroblast (L929 cells)	[88]
2021 *Origanum vulgare* Oregano	carvacrol (>80.00%)	PLCL/hyaluronic acid (MW = 100,00–20,000 Da)/ZnO nanoparticle (size ≤ 40 nm)	Nanofibers	Turned out to be biocompatible, antioxidant, anti-inflammatory, and antibacterial; potential in epithelialization, granulation tissue formation, neo-vascularization, and collagen deposition	[402]
2021 *Zingiber cassumunar*	(*E*)-1-(3,4-dimethoxyphenyl) butadiene ^a^	PLA (Mw~60,000)/PEO (Mw~100,000)	Fibrous Membrane	Showed antibacterial activity against *S. aureus* and *E. coli*; exhibited no toxicity to both human fibroblast and keratinocyte cells	[132]
2022 *Cymbopogon citratus* Lemongrass	geranial or α-citral (47.03%) and neral or β-citral (41.11%)	CS (molar mass~1.47 × 105 g/mol; degree of deacetylation~86.7%)	Films	Showed activity against *Escherichia coli* and *Staphylococcus aureus*; good antioxidant properties and non-toxicity	[403]
2022 *Cymbopogon flexuosus* Lemongrass	geraniol and citronellol ^a^	Collagen hydrolysate (bovine skin)/CS (highly viscose in the form of crystals, viscosity of 1267 Mpa.s)	Membranes	Antimicrobial efficiency against Gram-positive and Gram-negative bacteria and an opportunistic pathogenic yeast	[404]
2022 *Syzygium aromaticum* or *Eugenia caryophyllata* Clove	eugenol (85.00%), benzyl alcohol (34.00%), β-caryophyllene (0.30–13.00%), and eugenyl acetate (6.00%)	CS (low molecular weight, 50–190 kg/mol, degree of deacetylation: 75–85%)/gelatin (type A, porcine skin)	Scaffolds	Have biocompatibility, and antibacterial property	[405]
2022 *Origanum vulgare* Oregano	carvacrol ^a^	PVDF (Mw = 534,000)	Scaffolds’ Nanofiber	Biocompatibility in human normal cells; apoptosis-mediated anticancer activity was enhanced; showed good activity against the liver cancer cell line and triple-negative breast cancer cell line	[406]
2022 Eugenol	chemical composition not reported	PCL (molecular weight of 80,000, density of 1.145 g/cm^3^)	Nanofiber Scaffold	Non-toxic behavior and anti-adhesive properties against Gram-positive and Gram-negative bacteria	[93]
2022 Cinnamon					
2022 Frankincense	chemical composition not reported	Gelatin/Persian gum/bacterial nanocellulose	Film	Enhanced anti-inflammatory and antibacterial activity in the films; blood compatibility tests of the films showed no hemolytic nature	[407]
2022 *Teucrium polium* Halpa					
2022 *Thymus vulgaris* Thyme	carvacrol ^a^	κ-Carrageenan (Mw = 400–560 KDa)/PEG (powder, Mw = 35,000 g/mol)	Hydrogel Membranes	Showed > 95% antimicrobial activity against both Gram-positive and Gram-negative bacteria	[123]
2023 *Copaifera officinalis* Copaiba	β-caryophyllene (64.26%)	PCL (42,500 g/mol)/CS (low molecular weight, 190,000 g/mol, deacetylation degree of 85%)	Nanocapsules’ Film	Provided antibacterial activity against *Staphylococcus aureus* and *Pseudomonas aeruginosa*	[257]
2023 *Origanum vulgare* Oregano	thymol (37.72%), γ-terpinene (15.19%), isopropyl o-cresyl sulfide (14.19%), *trans*-caryophyllene (9.43%), linalool (5.51%), and β-myrcene (4.59%)	CS/PVP/gelatin (not mentioned information)	Hydrogel	Nanoemulgel assisted by low-level laser therapy facilitated wound healing, reduced inflammation, and enhanced granulation tissue and re-epithelialization	[346]
2023 *Cinnamomum cassia* bark Cinnamon	(*E*)-cinnamaldehyde (75.31%), (+)-3-carene (8.12%)	Cellulose nanofibers/CS (not mentioned information)	Aerogel Scaffolds	Exhibited more potent antibacterial activity against *S. aureus* and *E. coli* and enhanced biocompatibility; significantly enhanced the proliferation of L929 fibroblast cell	[408]
2023 *Origanum vulgare* Oregano	carvacrol ^a^	Bioactive glass: TEOS/TEP/PVP (Mw ¼ 130 kg/mol); nanofibrous scaffolds: gelatin (type B, 48,722-500G-F)/PLGA (Mw ¼ 95 kg/mol)	Bioactive Glass and Nanofibrous Scaffolds	Substantially improved wound re-epithelialization and neo-vessel formation, induced macrophage polarization, suppressed inflammation, and promoted scarless wound healing	[409]
2023 *Eugenia caryophyllata* and *E. aromaticum* Clove	eugenol (81.93%) and β-caryophyllene (12.28%)	Cellulose acetate (Mn = 30,000) Retinyl palmitate	Scaffolds	Was biocompatible with L929 fibroblast cells and had antibacterial and antioxidant properties	[410]

^a^: % not reported; CS: Chitosan; RI: Retention index; PLA: Poly(lactic acid); PVP: Poly(vinyl pyrrolidone); PVA: Poly(vinyl alcohol); PCL: Poly(ε-caprolactone); NHDF: Normal human dermal fibroblast; PU: Polyurethane; PEG: Polyethyleneglycol; PEO: Polyethylene oxide; PLCL: Poly(L-lactide-co-caprolactone); CMC: Carboxymethyl chitosan; CBM: Carbomer; PBS: Phosphate-buffered saline; PVDF: Poly(vinylidene fluoride); TEOS: Tetraethyl orthosilicate; TEP: Triethyl phosphate; PLGA: Poly(L-lactide-co-glycolide).

## Data Availability

The data presented in this study are available on request from the corresponding author.
